# Diagnosing Parkinson's Diseases Using Fuzzy Neural System

**DOI:** 10.1155/2016/1267919

**Published:** 2016-01-10

**Authors:** Rahib H. Abiyev, Sanan Abizade

**Affiliations:** ^1^Department of Computer Engineering, Applied Artificial Intelligence Research Centre, Near East University, Lefkosa, Northern Cyprus, Mersin 10, Turkey; ^2^Department of Electrical and Electronic Engineering, Applied Artificial Intelligence Research Centre, Near East University, Lefkosa, Northern Cyprus, Mersin 10, Turkey

## Abstract

This study presents the design of the recognition system that will discriminate between healthy people and people with Parkinson's disease. A diagnosing of Parkinson's diseases is performed using fusion of the fuzzy system and neural networks. The structure and learning algorithms of the proposed fuzzy neural system (FNS) are presented. The approach described in this paper allows enhancing the capability of the designed system and efficiently distinguishing healthy individuals. It was proved through simulation of the system that has been performed using data obtained from UCI machine learning repository. A comparative study was carried out and the simulation results demonstrated that the proposed fuzzy neural system improves the recognition rate of the designed system.

## 1. Introduction

In the world, many people suffer from Parkinson's disease (PD). The disease more often appeared after the age of 60 [1]. Parkinson's disease is a chronicle disorder of central nervous system which causes the death of the nervous cell in the brain. Parkinson's disease is progressive and the number of people suffering from the disease is expected to rise. The disease usually happens slowly and persists over a long period of time.

The symptoms of the PD continue and worsen over time. The basic symptoms of PD are movement related symptoms. These are tremor, rigidity or stiffness of the limbs and trunk, bradykinesia or slow movement, and problems with balance or walking [2, 3]. Tremor is a basic symptom which may affect shaking or trembling of legs, arms, hands, jaw, or face. The patients may have difficulty talking, walking, or completing some other simple tasks as these symptoms become more pronounced. Other symptoms are related to the behavioural problems, depression, thinking, sleep, and emotional problems. A person with Parkinson's may have a trouble in speaking and swallowing and chewing problems. Especially in advanced stages of the disease nonmotor features, such as dementia and dysautonomia, occur frequently. The diagnosis and timely treatments are important in order to manage its symptoms. The diagnosis is based on neurological examination and medical history of patients. The diagnosis of the disease in the early stages is difficult [3]. Diagnosis of PD depends on the presence of two or more of the above symptoms.

Vocal symptoms that include impairment of vocal sounds (*dysphonia*) and problems with the normal articulation of speech (*dysarthria*) are important in diagnosis of PD [4]. The research paper [5] shows that the most important symptom of PD is dysphonia. The dysphonia is the disorder of voice. Dysphonic symptoms typically include reduced loudness and roughness and breathiness and decreased energy in the higher parts of the harmonic spectrum and exaggerated vocal tremor. The treatment of these symptoms is difficult for the people having Parkinson's disease. In [4–6] it was shown that approximately 90% of people with Parkinson's disease have dysphonia. Dysphonia includes any pathological or functional problem with voice [6]. The voice will sound hoarse, strained, or effortful. It may be difficult to understand the voice of people having PD. The used method for diagnosis of Parkinson's disease (PD) is basically based on speech measurement for general voice disorders [4, 7–9].

Specialists doctors need to make an analysis of many factors for accurate diagnose of PD. Usually, decisions made are based on evaluating the current test results of patients. The problem becomes too difficult if the number of attributes that the specialist wants to evaluate is high. Recently various computational tools have been developed in order to improve the accuracy of diagnosis of PD. These tools have provided excellent help to the doctors and medical specialists in making decisions about the patients. Different Artificial Intelligence (AI) techniques, expert systems, and decision making systems are designed for diagnosis or classification of diseases. They were potential and good supportive tools for the expert/doctor. The development of efficient recognition systems in medical diagnosis is becoming more important. Nowadays various Artificial Intelligence techniques such as expert systems, fuzzy systems, and neural networks are actively applied for diagnosis of Parkinson's diseases using voice signals. Reference [4] introduces a new measure of dysphonia, pitch period entropy (PPE), which is robust to many uncontrollable confounding effects including noisy acoustic environments and separates healthy people from the people having PD. Nonlinear dynamical systems theory [4, 10] and statistical learning theory, such as* linear discriminant analysis *(LDA) and* support vector machines *(SVMs) [5, 11], are preferred for classification of healthy people or those with PD and discriminate the healthy people on the basis of measures of dysphonia. Different techniques, such as SVM [12], SVM with RBF (radial based function) kernel [13], SVM with Multiple Layer Perceptron (MLP), and a Radial Basis Function Network (RBFN) [14], are used for diagnosis of PD. In [15] integration of Kohonen self-organizing map (KSOM) and least squares support vector machine (LS-SVM), and in [3, 16] nonlinear time series analysis tools are applied for diagnosing of PD. Reference [17] uses fuzzy c-means algorithm, [18] uses four independent classification schemas, neural networks, DMneural, regression, and decision tree for classification purpose, and a comparative study was carried out.

The above methods are used in order to increase classification accuracy of PD. Classification systems can help in increasing the accuracy and reliability of diagnoses and minimizing possible errors, as well as making the diagnoses more time efficient. Success in the discovery of knowledge depends on the ability to explore different classes of specific data and to apply appropriate methods in order to extract the main features. This paper deals with the application of fusion of fuzzy systems and neural networks for designing of the recognition system of PD.

Fuzzy systems can handle uncertainties associated with information or data in the knowledge bases [19] and are widely used to solve different real world problems. Fuzzy system uses data and knowledge specific to chaotic dynamics of the process and increases the performance of the system. In the literature, different neural and fuzzy structures are proposed for solving various problems [20–26]. In [22, 23] clustering algorithm and gradient descent algorithm are applied for the design of multi-input and single output FNS. Well known ANFIS (adaptive neurofuzzy inference system) structure is used for solving cervical cancer recognition [27], for optimizing the chiller loading [28], and for distinguishing ESES (electrical status epilepticus) and normal EEG (electroencephalography) signals [29]. The use of multiple ANFIS structures, in [27], leads to the increase of the number of parameters of the network. In these papers the used systems are designed for special purpose and most of them are basically based on Mamdani type rules. Performances of these systems are determined by measuring classification rate. In this paper, in order to improve the performance of classification system, a multi-input and multioutput fuzzy neural system (FNS) based on TSK rules is proposed for identification of the PD.

The paper is organized as follows.  Section 2 describes the structure of proposed fuzzy neural system used for recognition of PD. The parameter update rule of the proposed system is presented in Section 3.  Section 4 describes the simulation results. The conclusions are given in Section 5.

## 2. FNS Based Recognition 

The fuzzy neural system combines the learning capabilities of neural networks with the linguistic rule interpretation of fuzzy inference systems. The design of FNS includes the development of the fuzzy rules that have if-then form. This can be achieved by dint of optimal definition of the premise and consequent parts of fuzzy if-then rules for the classification system through the training capability of neural networks. The two basic types of if-then rules used in fuzzy systems are Mamdani and Takagi-Sugeno-Kang (TSK) type fuzzy rules. The first type consists of rules, whose antecedent and consequent parts utilize fuzzy values. The second one uses the fuzzy rules that have fuzzy antecedent and crisp consequent parts. In the paper we use TSK type fuzzy rules for system design. The second type of fuzzy system approximates nonlinear system with linear systems and has the following form:

(1) If  x1  is  A1j  and  x2  is  A2j  and  …  and  xm  is  AmjThen  yj  is  ∑i=1maijxi+bj,

where *x*_*i*_ and *y*_*j*_ are input and output signals of the system, respectively, *i* = 1,…, *m* is the number of input signals, and *j* = 1,…, *r* is the number of rules. *A*_*ij*_ are input fuzzy sets; *b*_*j*_ and *a*_*ij*_ are coefficients.

**Figure 1:**
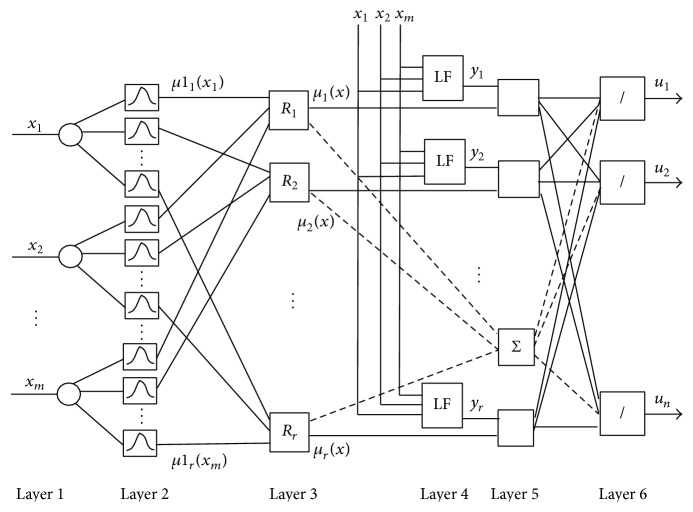
Classifier based on FNS.

The structure of fuzzy neural networks used for the classification of PDs is based on TSK type fuzzy rules and is given in Figure 1. The FNS includes six layers. In the first layer, *x*_*i*_  (*i* = 1,…, *m*) input signals are distributed. The second layer includes membership functions. Here each node corresponds to one linguistic term. Here, for each input signal entering the system, the membership degree to which input value belongs to a fuzzy set is calculated. The Gaussian membership function is used in order to describe linguistic terms:

(2) $$\begin{array}{c} \mu 1_{j}^{}\left(\;x_{i}\;\right)=e^{-{\left(x_{i}-c_{ij}\right)}^{2}/\sigma_{ij}^{2}},\;i=1,\ldots ,m,\;\;j=1,\ldots ,r, \end{array}$$

where *c*_*ij*_  and  *σ*_*ij*_ are center and width of the Gaussian membership functions, respectively, and *μ*1_*j*_(*x*_*i*_) is membership function of *i*th input variable for *j*th term.

The third layer is a rule layer. Here number of nodes is equal to the number of rules. Here *R*_1_, *R*_2_,…, *R*_*r*_ represents the rules. The output signals of this layer are calculated using t-norm min (AND) operation:

(3) $$\begin{array}{c} \mu_{j}^{}\left(\;x\;\right)=\prod_{i}\mu 1_{j}\left(\;x_{i}\;\right)\;i=1,\ldots ,m,\;\;j=1,\ldots ,r, \end{array}$$

where Π is the min operation.

These *μ*_*j*_(*x*) signals are input signals for the fifth layer. Fourth layer is a consequent layer. It includes *n* linear systems. Here the output values of the rules are determined using linear functions (LF):

(4) $$\begin{array}{c} y1_{j}=\overset{m}{\underset{i=1}{\sum }}x_{i}w_{ij}+b_{j}. \end{array}$$

In the fifth layer, the output signals of the third layer are multiplied by the output signals of the fourth layer. The output of *j*th node is calculated as *y*_*j*_ = *μ*_*j*_(*x*) · *y*1_*j*_.

The output signals of FNS are determined as

(5) $$\begin{array}{c} u_{k}=\frac{\sum_{j=1}^{r}w_{jk}y_{j}}{\sum_{j=1}^{r}\mu_{j}\left(x\right)}. \end{array}$$

Here *u*_*k*_ are the output signals of FNS (*k* = 1,…, *n*) and *w*_*jk*_ are weight coefficients of connections used between layers 5 and 6. After calculating the output signal, the training of the network starts.

## 3. Parameter Updates

### 3.1. Fuzzy Classification

The design of FNS (Figure 1) includes determination of the unknown parameters of the antecedent and the consequent parts of the fuzzy if-then rules (1). In fuzzy rules the antecedent part represents the input space by dividing the space into a set of fuzzy regions and the consequent part describes the system behaviour in those regions.

As mentioned above, recently a number of different approaches have been used for designing fuzzy if-then rules. Some of them are based on clustering [20–24, 26], the least squares method (LSM) [20, 22, 30], gradient algorithms [14, 20–23, 26], genetic algorithms [24, 25, 28], and particle swarm optimization (PSO) [31].

In this paper, fuzzy clustering and gradient technique are used for the design of FNS. At first the fuzzy clustering is used to design the antecedent (premise) parts, and then gradient algorithm is used to design the consequent parts of the fuzzy rules. Fuzzy clustering is an efficient technique for constructing the antecedent structures. The aim of clustering methods is to identify a certain group of data from a large data set, such that a concise representation of the behaviour of the system is produced. Each cluster center can be translated into a fuzzy rule for identifying the class. Different clustering algorithms are developed [32–34]. Recently fuzzy c-means [32] and subtractive clustering [33, 34] algorithms have been developed for fuzzy systems. Subtractive is unsupervised clustering [33] which is an extension of the grid based mountain clustering [34]. Here the number of clusters for input data points is determined by the clustering algorithm. Sometimes we need to control the number of clusters in an input space. In these cases, the supervised clustering algorithms are of primary concern. Fuzzy c-means clustering is one of them. It can efficiently be used for fuzzy systems [32] with a simple structure and sufficient accuracy. In this paper, the fuzzy c-means (FCM) clustering technique is used for structuring the premise part of the fuzzy system.

Learning of FNS starts with the update of parameters of antecedent part of if-then rules, that is, the parameters of the second layer of FNS. For this aim FCM classification is applied in order to partition input space and construct antecedent part of fuzzy if-then rules. The following objective function is used in FCM algorithm:

(6) $$\begin{array}{c} J_{q}=\overset{N}{\underset{i=1}{\sum }}\overset{C}{\underset{j=1}{\sum }}u_{ij}^{q}d_{ij}^{2},\;where\;\;d_{ij}=\left\|x_{i}-c_{j}\right\|,\;\;1\leq q<\infty , \end{array}$$

where *q* is any real number greater than 1, *u*_*ij*_ is the degree of membership of *x*_*i*_ in the cluster *j*, *x*_*i*_ is the *i*th of *d*-dimensional measured data, *c*_*j*_ is the *k*-dimension center of the cluster, and ‖*∗*‖ is any norm expressing the similarity between any measured data and the cluster centers.

The fuzzy classification of input data is carried out through an iterative optimization of the objective function (6), with the update of membership *u*_*ij*_ and the cluster centers *c*_*j*_. The algorithm consists of the following steps:

(1) Initialize *U* = [*u*_*ij*_] matrix, *U* ^(0)^.
(2) Calculate the centers vectors *C* ^(*t*)^ = [*c*_*j*_] with *U* ^(*t*)^:
(7) $$\begin{array}{c} c_{j}=\frac{\left(\sum_{i=1}^{N}u_{ij}^{q}\cdot x_{i}\right)}{\sum_{i=1}^{N}u_{ij}^{q}}. \end{array}$$
(3) Update *U* ^(*t*)^ and *U* ^(*t*+1)^:
(8) $$\begin{array}{c} u_{ij}=\frac{1}{\sum_{k=1}^{C}{\left(d_{ik}^{}/d_{jk}^{}\right)}^{2/\left(q-1\right)}}. \end{array}$$
(4) If {|*U* ^(*t*+1)^ − *U* ^(*t*)^|} < *ε* then stop; otherwise set *t* = *t* + 1 and return to Step (2).

In the results of partitioning the cluster centers are determined. These cluster centers will correspond to the centers of the membership functions used in the input layer of FNS. The width of the membership function is determined using the distance between cluster centers.

After the design of the antecedents parts by fuzzy clustering, the parameter update rules are derived for training the parameters of the consequent parts of the fuzzy rules. In the paper, we applied gradient learning with adaptive learning rate. The adaptive learning rate guarantees the convergence and speeds up the learning of the network.

### 3.2. Learning Using Gradient Descent

At the beginning, the parameters of the FNS are generated randomly. To generate a proper FNS model, the training of the parameters has been carried out. For generality, we have given the learning procedure of all parameters of FNS using gradient descent algorithm. The parameters are the membership function of linguistic values in the second layer of the network and the parameters of the fourth and fifth layers. In the design of FNS cross validation technique is used for separation of the data into training and testing set. Training includes the adjusting of the parameter values. In this paper, a gradient learning with adaptive learning rate is applied for the update of parameters. The adaptive learning rate guarantees the convergence and speeds up the learning of the network. In addition, the momentum is used to speed up the learning processes.

The error on the output of the network is calculated as

(9) $$\begin{array}{c} E=\frac{1}{2}\overset{n}{\underset{k=1}{\sum }}{\left(\;u_{k}^{d}-u_{k}\;\right)}^{2}. \end{array}$$

Here *n* is the number of output signals of the network, *u*_*k*_^*d*^  and  *u*_*k*_ are desired and current output values of the network (*k* = 1,…, *n*), respectively. The parameters *w*_*jk*_, *a*_*ij*_, *b*_*j*_ (*i* = 1,…, *m*, *j* = 1,…, *r*, *k* = 1,…, *n*) in consequent part of network and the parameters of membership functions *c*_*ij*_  and  *σ*_*ij*_ (*i* = 1,…, *m*, *j* = 1,…, *r*) in the premise part of FNS are adjusted using the following formulas:

(10) wjkt+1=wjkt−γ∂E∂wjk+λwjkt−wjkt−1;aijt+1=aijt−γ∂E∂aij+λaijt−aijt−1;bjt+1=bjt−γ∂E∂bj+λbjt−bjt−1;

(11) cijt+1=cijt−γ∂E∂cij+λcijt−cijt−1;σijt+1=σijt−γ∂E∂σij+λσijt−σijt−1;i=1,…,n;  j=1,…,r;  k=1,…,n.

Here *γ* is the learning rate, *λ* is the momentum, *m* is the number of input signals of the network (input neurons) and *r* is the number of fuzzy rules (hidden neurons), and *n* is the number of output neurons.

The derivatives in (10) are computed using the following formulas:

(12) ∂E∂wjk∂E∂uk∂uk∂wjk=ukt−ukdt·y1j∑j=1nμj,∂E∂aij∂E∂uk∂uk∂y1j∂y1j∂yj∂yj∂aij=∑kukt−ukdt·wkjμjxi∑j=1nμj,∂E∂bj∂E∂uk∂uk∂y1j∂y1j∂yj∂yj∂bj=∑kukt−ukdt·wkjμj∑j=1nμj,here  i=1,…,m,  j=1,…,r,  k=1,…,n.

The derivatives in (11) are determined by the following formulas:

(13) ∂E∂cij=∑k∂E∂uk∂uk∂μj∂μj∂cij,∂E∂σij=∑k∂E∂uk∂uk∂μj∂μj∂σij.

Here *i* = 1,…, *m*, *j* = 1,…, *r*, *k* = 1,…, *n*. Consider

(14) ∂E∂uk=ukt−ukdt;∂uk∂μj=yj−uk∑j=1nμj;∂μjxi∂cij=μjxi2xi−cijσij2;∂μjxi∂σij=μjxi2xi−cij2σij3.

Using equations (12)–(14), the derivatives in (10) and (11) are calculated and the correction of the parameters of FNS is carried out.

Convergence is very important problem in learning of FNS model. The convergence of the learning algorithm using gradient descent depends on the selection of the initial values of the learning rate. Usually, the initial value of learning rate is selected in the interval [0-1]. A large value of the learning rate may lead to unstable learning; a small value of the learning rate results in a slow learning speed. In the paper an adaptive approach is applied for updating these parameters. The learning of the FNS parameters is started with a small value of the learning rate *γ*. During learning, *γ*is increased if the value of change of error Δ*E* = *E*(*t*) − *E*(*t* + 1) is positive and decreased if negative. This strategy ensures a stable learning for the FNS. In addition a momentum term is used to speed up learning processes. The optimal value of the learning rate for each time instance can be obtained using a Lyapunov function [22, 23]. The derivation of the convergence is given in [22, 23].

## 4. Simulation Studies

The FNS, described above, is applied for classification of Parkinson's dieses. The people are divided into two classes: normal and PD. For this aim, the database is taken from University of California at Irvine (UCI) machine learning repository. The data set is donated from hospitals and it has been studied by many researchers. The data set includes biomedical voice measurements of 31 people; 23 were diagnosed with PD. Each row contains the value of the 23 voice parameters. Each column contains 195 items of data for each parameter. The main aim of the data is to discriminate healthy people from those with PD. The parameters that are used for recognition of PD are given in Table 1. These are the parameters of the voice signals recorded directly on the computer using Computerized Speech Laboratory. During modelling the preprocessing have been done on the input data and the input data are normalized in the interval of [0,1]. The scaling operation helps and makes the training process of the system easy. After normalization, these data are entered as an input signal to the FNS.

To design classification model the FNS structure with 23 input and 2 output neurons is generated first. If we use traditional neurofuzzy structure (e.g., [20] or [26]) for 23 inputs and 2 cluster centers, pow(2,23) = 8383608 rules should be generated. The rules are constructed using all possible combinations of inputs and cluster centers. This is very large number. In this paper the number of rules is selected according to the clustering results, equal to cluster centers.

In the design of FNS, the fuzzy classification is applied in order to partition input space and select the parameters of the premise parts, that is, the parameters of Gaussian membership functions used in the second layer of FNS. FCM clustering is used for the input space with 16 clusters for each input. 16 fuzzy rules are constructed using a different combination of these clusters for 22 inputs. After clustering input space gradient decent algorithm is used for learning of consequent parts of the fuzzy rules, that is, parameters of the 4th layer of FNS. Learning is implemented using cross validation. Cross validation generalizes two independent data sets: training and testing. It is applied to find accurate model of classifier. In the paper 10-fold cross validation is used for separation of the data into training and testing set and for evaluation of classification accuracy. There should be set of experiments in order to achieve required accuracy in the FNS output. The simulation is performed using different number of neurons in hidden layer. The design steps of FNS for the diagnosing PD are given below:

1. Read PD data set. Select input and output (target) signals from statistical data. Apply normalization.
2. Enter the values of learning rate and momentum. Set the number of clusters. Generate network parameters. Set a maximal number of epochs for learning.
3. Apply classification algorithm to the input signals and determine the cluster centers.
4. Use cluster centers to determine the centers of membership functions of layer 2.
5. Use the centers of membership functions to determine the widths of membership functions.
6. Using input statistical data define a random partition for 10-fold cross validation.
7. Initialize current number of learning epochs to 1.
8. Use PD data set and cross validation and determine training and testing data sets.
9. Determine the numbers of rows in training and testing data sets.
10. Initialize the number of iterations to 1.
11. According to the number of iterations select input data from training data set and send them to the input of FNS.
12. Calculate network outputs.
13. Determine the values of errors using network output and target output signals. Use these error values to compute the sum of the squared errors (SSE).
14. Using error values update the network parameters (learning of network).
15. Apply adaptive strategy for updating the learning rate using current and previous values of SSE.
16. Compute sum of SSE obtained on each iteration and save as the training error. Repeat Steps (11)–(16) for other remaining training data sets. If the current number of iterations will be less than a number of rows in the training set then go to Step (11), otherwise go to Step (17).
17. Select test data set.
18. Set number of iterations to 1.
19. According to the number of iterations select input data from test data set and send them to the input of FNS.
20. Compute the output of FNS.
21. Determine the values of errors using network output and target output signals. Compute SSE on the output of the network.
22. Compute sum of SSE obtained on each iteration of the loop and save as the testing error. Repeat Steps (19)–(22) for other remaining test data sets.
23. Check the value of testing error with the value of testing error obtained in the previous epoch. If the current error value is less than the previous one then go to Step (24), otherwise go to Step (25).
24. Save the parameters of the network. Save the values of training and testing errors.
25. Use the sum of SSE to find root mean squared error (RMSE). Print the values of testing and training errors; increment the epochs number.
26. Check a current number of epochs for the continuation of the learning process. If this number is less than the maximal number of epochs then repeat Steps (8)–(26). Otherwise go to Step (27).
27. Print the values of training and testing errors obtained in Step (24).
28. Stop the training.

The training of input/output data for the classification system will be a structure whose first component is the twenty-three-dimension input vector and second component is the two-dimension output clusters.  Table 2 depicts the fragment from PD data set. The FNS structure is generated with 23 input and two output neurons. After generation fuzzy c-means clustering and gradient descent algorithms are applied for training the parameters of FNS. In the first step, using fuzzy clustering, cluster centers are determined using the input data. These cluster centers are used to organize the membership functions of the inputs of antecedent part of each fuzzy rules. The rule layer is the second layer. The consequent parts of the fuzzy rules are organized using linear functions. Linear functions are determined in fourth layer. After clustering and designing antecedent part the learning of the parameters of consequent part starts. The initial values of the parameters *w* and *b* of linear functions of consequent part are selected in interval [0,0.2]. The initial values of learning rate and momentum are selected as 0.02 and 0.625, correspondingly. During learning the parameters *w* and *b* of the rule are updated. In the results of learning the fuzzy rules are constructed. The clusters obtained from classification operation will be the centers of Gaussian membership functions used in antecedent parts of fuzzy rules. The consequent parts are constructed on the basis of learning of the parameters of linear functions.

The simulation results of FNS is compared with the simulation results of other models used for classification of PD. For evaluation of the outcomes of the models the Root Mean Square Error (RMSE) is used:

(15) $$\begin{array}{c} RMSE=\sqrt{\frac{1}{N}\overset{N}{\underset{i=1}{\sum }}{\left(u_{i}^{d}-u_{i}^{}\right)}^{2}}. \end{array}$$

Here *u*_*i*_^*d*^ are desired values of output and *u*_*i*_ are actual values of the system output.

To estimate the performance of the FNS clustering systems, the recognition rates and RMSE values of errors between clusters and current output signal are taken. RMSE is computed using formula given above. Recognition rate is computed by the number of items correctly classified divided by the total number of items:

(16) Recognition_rate=Number  of  items  correctly  classifiedTotal  number  of  items·100%.

**Figure 2:**
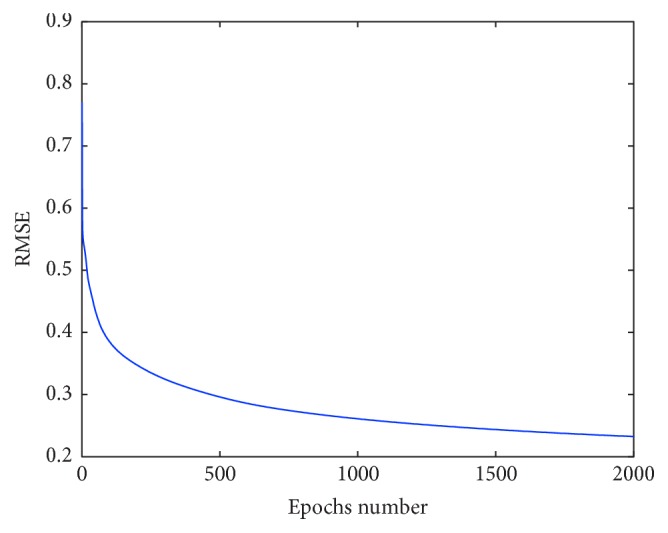
RMSE values.

During training of FNS, all input data are scaled to interval [0,1]. Then fuzzy c-means clustering is applied to input data. The result of clustering is used to set up parameters of the antecedent part of fuzzy rules, that is, parameters of the second layer of FNS structure. The parameters of the consequent part of fuzzy rules are determined by applying gradient learning. The learning has been performed for 2000 epochs. The synthesis of FNS classification system is performed using different number of fuzzy rules. The training has been performed using different number of rules: 2, 5, 8, 12, and 16. Training is performed using 10-fold cross validation. In the results of training the parameters of FNS are determined.  Figure 2 depicts the values of RMSE obtained during training. Once the FNS is trained then it has been used for testing. The values of RMSE obtained for train, evaluation, and test stages for FNS having 16 hidden neurons are 0.232154, 0.291636, and 0.283590, correspondingly. The training has been performed with the learning rate 0.01 and momentum rate 0.825.  Table 3 describes training and testing results of FNS model obtained using different number of rules: 2, 5, 8, 12, and 16. Simulation results are averaged over ten simulations.

From Table 3, it was shown that the increase in the number of rules (or the number of hidden neurons) decreases the values of RMSE for training and testing cases and increases recognition rate. The use of clustering and gradient techniques for learning allows quick obtaining of low RMSE value and allows improving performance of FNS for training and testing stages. In the second simulation a comparative analysis of the classification of PD has been performed. The result of the simulation of the FNS classification model is compared with results of simulations of different classification models, such as support vector machine (SVM), neural networks (NN), regression model, decision tree, and FCM based feature weighting. To estimate the performance of the NN, SVM, and FNS clustering systems, the recognition rates and RMSE values of errors between clusters and current output signal are compared. In Table 4, the comparative results of simulations of different models are given. As shown in the table the performance of FNS classification system is better than the performance of the other models.

## 5. Conclusion

The paper presents the diagnosis of Parkinson's diseases using fuzzy neural structures. The structure and learning algorithms of FNS are presented. Fuzzy clustering and gradient descent learning algorithms are applied for the development of the FNS. Learning is performed using 10-fold cross validation data set. The design of the classification system is carried out using different number of fuzzy rules used in FNS. Recognition rate of classification is obtained as 100% with 16 hidden neurons. For comparative analysis, the simulation of PD is performed using different models. The obtained results demonstrate that the performance of FNS is better than the other models used for classification of PD.

## Figures and Tables

**Table 1:** List of measurement methods applied to acoustic signals recorded from each subject.

Name	ASCII subject name and recording number
MDVP:Fo (Hz)	Average vocal fundamental frequency
MDVP:Fhi (Hz)	Maximum vocal fundamental frequency
MDVP:Flo (Hz)	Minimum vocal fundamental frequency
MDVP:Jitter (%)	Five measures of variation in fundamental frequency
MDVP:Jitter (Abs)	
MDVP:RAP	
MDVP:PPQ	
Jitter:DDP	
MDVP:Shimmer	Six measures of variation in amplitude
MDVP:Shimmer (dB)	
Shimmer:APQ3	
Shimmer:APQ5	
MDVP:APQ	
Shimmer:DDA	
NHR	Two measures of ratio of noise to tonal components in the voice
HNR	
RPDE	Two nonlinear dynamical complexity measures
D2	
DFA	Signal fractal scaling exponent
Spread1	Three nonlinear measures of fundamental frequency variation
Spread2	
PPE	
Status	Health status of the subject: one, Parkinson's; zero, healthy

**Table 2:** Fragment from PD data set.

MDVP:Fo (Hz)	119.99200	122.4000	236.20000	237.32300	260.10500	197.56900	151.73700	148.7900
MDVP:Fhi (Hz)	157.30200	148.6500	244.66300	243.70900	264.91900	217.62700	190.20400	158.3590
MDVP:Flo (Hz)	74.99700	113.8190	102.13700	229.25600	237.30300	90.79400	129.85900	138.9900
MDVP:Jitter (%)	0.00784	0.00968	0.00277	0.00303	0.00339	0.00803	0.00314	0.00309
MDVP:Jitter (Abs)	0.00007	0.00008	0.00001	0.00001	0.00001	0.00004	0.00002	0.00002
MDVP:RAP	0.00370	0.00465	0.00154	0.00173	0.00205	0.00490	0.00135	0.00152
MDVP:PPQ	0.00554	0.00696	0.00153	0.00159	0.00186	0.00448	0.00162	0.00186
Jitter:DDP	0.01109	0.01394	0.00462	0.00519	0.00616	0.01470	0.00406	0.00456
MDVP:Shimmer	0.04374	0.06134	0.02448	0.01242	0.02030	0.02177	0.01469	0.01574
MDVP:Shimmer (dB)	0.42600	0.62600	0.21700	0.11600	0.19700	0.18900	0.13200	0.14200
Shimmer:APQ3	0.02182	0.03134	0.01410	0.00696	0.01186	0.01279	0.00728	0.00839
Shimmer:APQ5	0.03130	0.04518	0.01426	0.00747	0.01230	0.01272	0.00886	0.00956
MDVP:APQ	0.02971	0.04368	0.01621	0.00882	0.01367	0.01439	0.01230	0.01309
Shimmer:DDA	0.06545	0.09403	0.04231	0.02089	0.03557	0.03836	0.02184	0.02518
NHR	0.02211	0.01929	0.00620	0.00533	0.00910	0.01337	0.00570	0.00488
HNR	21.03300	19.08500	24.07800	24.67900	21.08300	19.26900	24.15100	24.41200
RPDE	0.414783	0.458359	0.469928	0.384868	0.440988	0.372222	0.396610	0.402591
D2	0.815285	0.819521	0.628232	0.626710	0.628058	0.725216	0.745957	0.762508
DFA	−4.813031	−4.075192	−6.816086	−7.018057	−7.517934	−5.736781	−6.486822	−6.311987
Spread1	0.266482	0.335590	0.172270	0.176316	0.160414	0.164529	0.197919	0.182459
Spread2	2.301442	2.486855	2.235197	1.852402	1.881767	2.882450	2.449763	2.251553
PPE	0.284654	0.368674	0.119652	0.091604	0.075587	0.202879	0.132703	0.160306
Status	1	1	0	0	0	0	1	1

**Table 3:** Simulation results of FNS.

Number of hidden neurons	RMSE training	RMSE evaluation	RMSE testing	Accuracy (%)
2	0.548520	0.560548	0.551954	81.025641
5	0.397395	0.401047	0.379963	93.333333
8	0.341242	0.435460	0.428456	95.897436
12	0.333357	0.343488	0.335679	97.948718
16	0.232154	0.291636	0.283590	100

**Table 4:** Comparative results of different models for classification of PD.

Models	Accuracy (testing)
Decision tree [18]	84.3
Regression [18]	88.6
DMneural [18]	84.3
Neural network [18]	92.9
FCM based feature weighting [17]	97.93
SVM	93.846154
FNS	100
